# Problematic use of the Internet is a unidimensional quasi-trait with impulsive and compulsive subtypes

**DOI:** 10.1186/s12888-019-2352-8

**Published:** 2019-11-08

**Authors:** Jeggan Tiego, Christine Lochner, Konstantinos Ioannidis, Matthias Brand, Dan J. Stein, Murat Yücel, Jon E. Grant, Samuel R. Chamberlain

**Affiliations:** 1 Monash Institute of Cognitive and Clinical Neurosciences, and School of Psychological Sciences, Monash University, Monash, Australia; 2 SA MRC Unit on Risk & Resilience in Mental Disorders, Department of Psychiatry, Stellenbosch University, Stellenbosch, South Africa; 3 Department of Psychiatry, University of Cambridge, Cambridge Peterborough NHS Foundation Trust, Cambridge, UK; 4 Department of General Psychology: Cognition and Center for Behavioral Addiction Research (CeBAR), University of Duisburg-Essen, Duisburg, Germany; 5 SA MRC Unit on Risk & Resilience in Mental Disorders, Department of Psychiatry, University of Cape Town, Cape Town, South Africa; 6 Department of Psychiatry, University of Chicago, Chicago, USA; 7 Department of Psychiatry, Addenbrookes Hospital, Box 189 Level E4, Cambridge, CB2 0QQ UK

**Keywords:** Internet, Young’s, Scales, Psychometric, Impulsivity, Compulsivity

## Abstract

**Background:**

Problematic use of the Internet has been highlighted as needing further study by international bodies, including the European Union and American Psychiatric Association. Knowledge regarding the optimal classification of problematic use of the Internet, subtypes, and associations with clinical disorders has been hindered by reliance on measurement instruments characterized by limited psychometric properties and external validation.

**Methods:**

Non-treatment seeking individuals were recruited from the community of Stellenbosch, South Africa (*N* = 1661), and Chicago, United States of America (*N* = 827). Participants completed an online version of the Internet Addiction Test, a widely used measure of problematic use of the Internet consisting of 20-items, measured on a 5-point Likert-scale. The online questions also included demographic measures, time spent engaging in different online activities, and clinical scales. The psychometric properties of the Internet Addiction Test, and potential problematic use of the Internet subtypes, were characterized using factor analysis and latent class analysis.

**Results:**

Internet Addiction Test data were optimally conceptualized as unidimensional. Latent class analysis identified two groups: those essentially free from Internet use problems, and those with problematic use of the Internet situated along a unidimensional spectrum. Internet Addiction Test scores clearly differentiated these groups, but with different optimal cut-offs at each site. In the larger Stellenbosch dataset, there was evidence for two subtypes of problematic use of the Internet that differed in severity: a lower severity “impulsive” subtype (linked with attention-deficit hyperactivity disorder), and a higher severity “compulsive” subtype (linked with obsessive-compulsive personality traits).

**Conclusions:**

Problematic use of the Internet as measured by the Internet Addiction Test reflects a quasi-trait - a unipolar dimension in which most variance is restricted to a subset of people with problems regulating Internet use. There was no evidence for subtypes based on the type of online activities engaged in, which increased similarly with overall severity of Internet use problems. Measures of comorbid psychiatric symptoms, along with impulsivity, and compulsivity, appear valuable for differentiating clinical subtypes and could be included in the development of new instruments for assessing the presence and severity of Internet use problems.

## Background

Since its development in the 1980s, the Internet has become a global technology and is now used by > 50% of the world’s population, with penetrance being particularly high in North America, Europe, and parts of Asia (World Bank Global Data, 2018). While the Internet offers many benefits, it is recognized that some users develop excessive use, referred to by the umbrella term ‘Problematic Use of the Internet’ (PUI). Gaming Disorder, a manifestation of PUI, is likely to be included in the International Classification of Diseases Version 11 (ICD-11), and Internet gaming disorder has been listed as being in need of further study in the Diagnostic and Statistical Manual Version 5 (DSM-5) [1]. PUI has important public health consequences [21]. For example, people with PUI exhibit elevated rates of psychiatric disorders, including anxiety disorders, impulse control disorders, attention-deficit hyperactivity disorder [ADHD], and addictions [e.g. gambling, alcohol]; and associated physical health issues such as obesity [12, 30, 65]. It should be noted that causality has not yet generally been established, but these above-noted associations highlight the need to better define and operationalize PUI.

PUI has been the subject of considerable interest and theoretical debate since being introduced as a topic of study over 20 years ago [27, 70]. The Internet may be viewed as a conduit through which individuals manifest discrete behavioral syndromes such as Gambling Disorder, Gaming Disorder, Compulsive Buying/Shopping Disorder, or Compulsive Sexual Behavior Disorder [8, 28, 80]. In the alternative, PUI may be a syndrome in its own right, particularly if different online activities co-occur together to similar degrees with increasing symptom severity [7, 82]. Network modeling found that the broad construct of Internet Addiction had high centrality parameters in the model examined; i.e. was statistically important in explaining several types of technology-related activity including gaming and cybersex [4]. We recently found that cognitive dysfunction associated with PUI did not differ as a function of whether samples had Gaming Disorder, or other types of activity, suggesting commonalities across different manifestations of problematic Internet-related behaviors [32].

A vital precursor to addressing key research goals in the field of PUI research is to develop optimized measurement tools that are validated across cultural settings. Partially due to differing theoretical conceptualizations of PUI, there has been a proliferation of measures based on different foundations [42]. Many measures of PUI have poor psychometric properties, and/or have received little independent validation [36]. The Internet Addiction Test (IAT) [80] is generally regarded as the most widely used instrument in both research and practice. The IAT was a reformulation of the original Diagnostic Questionnaire [81], which conceptualized PUI as sharing parallels with pathological gambling, at that time considered an impulse control disorder in the DSM (and now listed as a Substance Related and Addictive Disorder). The IAT has 20 items, each self-rated on a 5-point scale (1: never/rarely, through to 5: always).

In general, the IAT has demonstrated high internal consistency, reliability, construct validity, and criterion-related validity [24, 76–78]. However, issues with item redundancy, factor instability, arbitrary cut-off scores, and lack of cross-cultural validity have been reported [42, 54]. These problems may stem from issues with the instrument per se, but could also reflect limitations of existing studies, including theoretical assumptions, sample characteristics, and statistical approaches. Much of the existing research into PUI seems to be underpinned by the continuity hypothesis and liability-threshold models, which suggest that clinical manifestations of psychopathology represent the most extreme elevations of normally distributed latent traits [19, 34]. Current studies implicitly assume PUI is a continuous and normally distributed latent trait by applying statistical analyses to group data obtained from community-based samples. However, it has been suggested that many psychopathology constructs are ‘*quasi-traits*’ – unipolar dimensions in which meaningful variation in the severity of a clinical syndrome can only be found at one end of the spectrum, with the other end of the continuum capturing its absence [63]. Addictive disorders, in particular, are likely to represent quasi-traits (also ‘unipolar’ traits) [47]. It has been proposed that addiction may arise as a consequence of an accumulation of small increases across multiple risk mechanisms over time [46]. A latent trait that represents the accumulation of small multiplicative processes would be predicted to be log-normal, with a majority of the population exhibiting low levels of the trait and a smaller proportion of individuals exhibiting elevated levels of the trait and clinically-relevant manifestations of psychopathology [47]. These positively-skewed distributions are often observed in health assessment and clinical measurement research [63].

PUI may similarly be a quasi-trait, with one end of the spectrum representing a meaningful variation in symptom severity (i.e. ‘Problematic Users of the Internet’), and the other a relative absence of problematic use (i.e. ‘Non-Problematic Users of the Internet’). This could explain some of the inconsistent findings across studies examining PUI using the IAT. If PUI constitutes such a quasi-trait, IAT items may function differently in measuring the underlying trait of PUI between groups, a property called differential item functioning [72]. Participant samples combining Problematic Users of the Internet and Non-Problematic Users of the Internet could give rise to psychometric instability of the IAT, as well as inconsistent findings across studies comprising different proportions of these groups [15, 48]. From this perspective, identifying and differentiating those individuals with PUI from the rest of the population would facilitate better characterization of PUI, the pathogenetic mechanisms, as well as potential interventions. As a quasi-trait, it is also possible that PUI represents a unidimensional spectrum of liability for developing and experiencing problematic patterns of Internet use. From this perspective PUI, as measured by the IAT, would be uni-factorial with all items measuring an underlying liability spectrum, akin to results previously reported for alcohol addiction [41]. If PUI is a narrow, unidimensional trait similar to other psychopathology constructs, the multi-dimensional structure and factor instability of the IAT observed across previous studies may reflect local statistical dependencies between redundant items with similar wording or content [62].

The inconsistent findings observed across studies of PUI and the IAT may also be attributable to the existence of clinical subtypes. Clinical subtypes have been investigated in other psychiatric disorders, such as depression; with these subtypes largely being defined by symptom severity [74]. Theories have posited subtypes of behavioral addiction, with some cases characterized by anti-sociality and impulsivity, whilst others are more strongly determined by psychological distress or environmental factors [50]. Alternatively, some have suggested a temporal transition from impulsive, initially reward-driven addictive behaviors to engagement in addictive patterns that are compulsive and more severe with increasing chronicity (Everitt & Robbins, 2016 [22];). Thus, inconsistent findings regarding the PUI and IAT may also be an artefact of collapsing analyses across latent classes or clinical subtypes [15, 48, 63]. The inconsistencies in psychometric properties of the IAT across studies, partly due to assumptions of continuity, render conceptual, theoretical, and empirical integration of the PUI literature problematic. Therefore, the current study evaluated IAT data across two distinct geographical and cultural settings, aiming to: (i) test whether PUI is best defined as a unidimensional quasi-trait; and (ii) to identify whether PUI is a unitary phenomenon or can be defined by subtypes (based on online activities and clinical data). We used conventional factor analyses (including consideration of bifactor models) and Latent Class Analysis (LCA). Exploratory Factor Analysis (EFA) and Confirmatory Factor Analysis (CFA) were first used to determine if the factor structure of the IAT was stable across discrete groups of participants according to sex and ethnicity. This was to ensure that differences in item functioning and the latent structure of the IAT were not more parsimoniously explained by differences in observed sample characteristics rather than latent classes [15, 48, 63]. CFA was then used to evaluate the fit of a bifactor model to the IAT and to determine if PUI could be conceptualized as a unidimensional trait [59, 61]. LCA is a type of mixture modeling capable of differentiating clinical subtypes of a given condition based on patterns of item endorsements [20, 52]. Individuals identified may then be further differentiated according to the nature of their symptoms, psychiatric comorbidities, or demographic characteristics. It was hypothesized that analysis would support PUI as a latent quasi-trait, and that subtypes of PUI would be identified based on the co-occurrence of impulsive and compulsive symptoms, with the latter being expected to be associated with more extensive PUI problems (due to the theorized shift from impulsivity to compulsivity, with the latter being expected to reflect more ingrained problems). We further predicted that subtypes would not be identified based on the type and extent of online activities engaged in, suggesting commonality across different behaviors.

## Methods

### Participants

The recruitment methods and nature of the sample have been described in detail previously [31, 33]. In brief, the sample comprised adults, recruited for an online survey from two sites: Stellenbosch, South Africa (*N* = 1661), and Chicago, United States of America (*N* = 827). Solutions based on factor analysis and latent class analysis are data-dependent and can be sample-specific (e.g. [52]; Vandenberg, 2002). Additionally, questionnaire items may function differently in measuring the underlying construct of interest across different groups of participants (e.g. [72]). Recruitment of two samples from different geographic locations was undertaken to enable independent cross-validation of the results. Comparison across cultural and geographically diverse samples was particularly important given past criticisms of the IAT as lacking cross-cultural validity [42, 54]. Individuals at both sites were recruited using Internet advertisements. Survey data were stored separately from personally identifiable data, so that responses made could not be linked back to a specific individual. The research was approved by local ethics committees. Participants did not receive compensation for taking part but were entered into a random lottery (i.e. lucky prize draw) whereby five prizes were available, valued between $50 and $200 in USA and three prizes between ZAR250 and ZAR750 in South Africa. To preserve data anonymity, the list of lottery participants was kept unlinked to the survey data. The Stellenbosch sample were used as the exploratory sample for initial factor analysis and LCA because there were more participants and parameter estimates were expected to be more stable than in the smaller Chicago sample [37, 52].

### Measures

The online survey included demographic questions: age, sex, ethnicity, relationship status, and education level. The Internet Addiction Test (IAT) [81] was included as the main instrument of interest, which comprised 20 questions examining facets of PUI, each scored 1–5, yielding a total sum score of 20–100 (higher scores being indicative of more extensive Internet-related problems). We also included questions about time engaged in different types of online activity [33]: general surfing; Internet gaming; Online role playing games (RPG); Time wasters/skill games (i.e. Apps on iPod/iPad/cell phone, Tetris, Jewels); Online action multiplayer (i.e. Call of Duty, Gears of War); Online shopping; Auction websites (e.g. E-bay); Online gambling; Social networking; Online sports (i.e. Fantasy sports, ESPN); Pornography/Sex on Internet; Messaging/Blogging (i.e. AIM, Skype); and Streaming videos/media (e.g. YouTube, Hulu). For each category of online activity, the individual responded 0–5 in terms of the average total number of hours spent on the given activity per day: 0 = None, 1 = < 1 h, 2 = 1–3 h, 3 = 4–5 h, 4 = 6–8 h, 5 = > 8 h.

The survey included the following clinical measures of interest. We focused on particular comorbidities that we expected to be implicated in PUI based on the extant literature [11] whilst also bearing in mind the need for the survey to not be excessively long for participants. Several modules were included from the Mini International Neuropsychiatric Interview (MINI) [66] adapted for online use, to screen for probable Obsessive Compulsive Disorder (OCD), Generalized Anxiety Disorder (GAD), Social Anxiety Disorder (SAD), and Avoidant Personality Disorder (APD). The World Health Organization’s ADHD Rating Scale (ASRS v1.1 Part A) was included to measure ADHD symptoms [35]. Obsessive Compulsive Personality Disorder (OCPD) tendencies were quantified by using a tick-list of the DSM criteria. These clinical measures relating to impulsivity and compulsivity were included because they are strongly implicated in addictions, and a theorized progression from impulsive to compulsive with worsening illness.

### Statistical procedures

#### Exploratory factor analysis of the IAT

Exploratory factor analysis (EFA) was performed as a precursor to Confirmatory Factor Analysis (CFA) [25]. The purpose of this preliminary EFA was to discover the number and structure of the latent dimensions underlying the IAT in our sample using an empirically-driven approach, because of the factor instability, sample specificity, and broad range of models reported in the literature [42]. The EFA was restricted to Caucasian women (*n* = 689) in the Stellenbosch sample as an a priori selected calibration subsample, because they represented the largest group and would therefore provide the most stable parameter estimates for subsequent comparison across sex and ethnic groups using invariance testing [69]. EFA was performed in SPSS 23 using Maximum Likelihood extraction; the number of factors retained was determined by examination of the scree plot and interpretability of the pattern and factor correlation matrices, and oblique rotation (direct oblimin) was used as the factor rotation method [16].

### Confirmatory factor analysis and multigroup invariance testing

CFA was implemented to evaluate the empirical fit of the model suggested by the EFA solution in the calibration subsample of Caucasian women, as well as determine if the factor solution was invariant across sex and ethnicity in the Stellenbosch sample. CFA was performed in M*plus* 7.2 using the covariance matrix [51]. The Weighted Least Squares Means and Variance (WLSMV) adjusted estimator with Theta parameterization was used for analysing the ordered categorical response variables of the IAT [9, 51]. Post hoc model fitting was performed by freeing error covariances for estimation one at a time with reference to theoretical plausibility and modification indices [67]. All parameters were deemed significant when adjusted for multiple post hoc comparisons using the Benjamini-Hochberg False Discovery Rate (B-H FDR *q* = .05) [6]. Model fit was evaluated using a combination of fit indices, including the chi square (*χ*^2^) test statistic (*p* > .05 = exact fit), Root Mean Square Error of Approximation (RMSEA) (ε < .05 close approximate fit; ε = .05–.08 reasonable approximate fit; ε > 1.0 poor approximate fit), Comparative Fit Index (CFI) (≥ .90 = adequate fit; ≥ .95 = good fit), and Weighted Root Mean Residual (WRMR) (> .95 = good fit). The chi square test statistic is overly sensitive to minor model misspecification in large samples (*N* > 300), thus approximate fit instances were used to adjudge model fit [37]. Invariance testing was used to compare the equivalence of factor structure, item loadings and thresholds, latent means, as well as factor variances and covariances across groups based on sex and ethnicity [9, 37, 75]. Partial invariance was considered where full invariance did not apply [10]. Due to the large sample size, small changes in the CFI (ΔCFI < .010) and RMSEA (ΔRMSEA < .015) were used to adjudge multigroup invariance instead of the more stringent *χ*^2^ chi square difference test [14, 49].

### Unidimensionality analyses

Unidimensionality of the IAT was assessed using a combination of metrics. First, the discriminant validity of the latent variables in the initial two-factor model solution in the Stellenbosch sample (see Results) was evaluated by calculating the average variance extracted (AVE) and the squared multiple correlation (SMC) [29, 37]. The SMC quantifies the amount of common variance shared between the factors, whereas the AVE quantifies the amount of variance explained by the factors across their indicators (i.e. IAT items). If the SMC is greater than the AVE the factors share more variance than they explain in their indicators suggesting they do not have discriminant validity and are better represented as a single construct [29]. Bifactor modeling was also used to evaluate the hypothesized unidimensionality of the IAT [59, 61]. The fit of the bifactor model to the IAT data in the Stellenbosch sample was first evaluated using conventional fit statistics. Unidimensionality statistics, including the explained common variance (ECV) and percentage of uncontaminated correlations (PUC), were then calculated using the standardized factor loadings and model parameters [59]. The ECV is a ratio of total item variance explained by the general factor compared to the residual group factors, with higher values indicating that more variance in the measure is explained by a common factor [59]. The PUC is the ratio of the number of item correlations within group factors compared to the total number of correlations [59]. When the ECV and PUC are both high (≥.70), measures can be collapsed into unidimensional constructs (i.e. total IAT raw scores) without introducing parameter bias [64].

#### Latent class analysis of the IAT

This approach was used first for the Stellenbosch dataset, and then for the Chicago dataset. Latent Class Analysis (LCA) was performed in Mplus 7.2 [51]. Procedures for class enumeration were consistent with recommendations provided by Nylund et al. [53] and Asparouhov & Muthén (2012) (see Additional file 1: Methods Detail A). The number of latent classes was assessed using a combination of fit statistics, including − 2*log-likelihood (− 2*LL), entropy (*E*), Bayesian Information Criterion (BIC), and the Lo–Mendell–Rubin (LMR) adjusted Likelihood Ratio Test (LRT) [53]. The combination of these statistics has been determined to provide a relatively sensitive measure of the true number of classes [53]. A non-significant *p* value for the LMR adjusted LRT indicates that the *k* – 1 class model provides a statistically better fit to the data than the *k* model or any subsequent *k* + 1 models [53]. Conversely, a significant *p* value indicates that *k* – 1 class model provides a statistically worse fit to the data than the *k* model or any subsequent *k* - 1 models. Simulations have shown that the Bootstrapped Likelihood Ratio Test (BLRT) is sensitive to inclusion of covariates in mixture modeling and performs less reliably when class separation (i.e. entropy) is high (*E* ~ 90) [17]. As the LCA conducted in the present study included covariates and produced results with high entropy, the BLRT was not examined. Online activities and clinical data (e.g. MINI modules) were included in the initial LCA as auxiliary variables for comparison across latent classes as distal outcome variables using the DCAT procedure described by Lanza et al. [44] and Asparouhov and Muthen (2014). This method avoids biased estimates in class comparisons, whilst preserving uncertainty in class membership without causing shifts in latent classes [2, 44]. The BCH method [5] was implemented for comparison of continuous distal outcome variables (age and IAT total scores) across classes as per current recommendations [3]. Where class separation was excellent (*E* > .9) between participants with and without PUI, latent class membership was assigned based on the posterior probabilities and was treated as a discrete observed variable. This enabled implementation of a within-class secondary LCA model for defining subtypes based on online activity and clinical measures in participants defined as PUI from the first LCA.

## Results

The combined participant sample comprised a total of 2488 individuals, *N* = 1661 from Stellenbosch, South Africa (*n* = 1020 female; age 18–88; *M* = 26.13; *SD* = 11.32) years and *N* = 827 from Chicago (USA) (*n* = 582 female; age 18–77; *M* = 35.83; *SD* = 14.42). Detailed sample characteristics are displayed in Additional file 1: Tables S1 – S3.

### EFA and CFA of the IAT

Exploratory Factor Analysis (EFA) in the calibration Stellenbosch subsample (women only) yielded an optimal two-factor solution for the IAT; explaining 48% of variance in responses. The model was estimated in M*plus* using CFA. IAT Item 5 failed to demonstrate statistically significant fit and was removed from the subsequent analysis. The final 2-factor model provided a reasonable overall fit (χ^2^(139) = 631.052, *p* < .001; RMSEA = .072 [90%*CI* = .066–.077]; CFI = .951; WRMR = 1.417) and is displayed in Additional file 1: Figure S1. Item loadings were generally high with no cross-loadings. The factor intercorrelation was also strong and statistically significant (ϕ = .803, *p* < .001). This two-factor IAT solution was replicated in males using CFA; as well as in further analyses restricted to Caucasian and non-Caucasian males and females, with invariance testing (Additional file 1: Table S4). These results indicated that the factor structure of the IAT was stable across sex and ethnicity in the Stellenbosch sample.

Formal invariance testing could not be performed across the Stellenbosch and Chicago samples, nor within the Chicago sample across subgroups (i.e. sex, ethnicity) because not all items categories were endorsed across groups leading to differences in threshold parameters. Therefore, a CFA was performed in the Chicago sample to determine if the same two-factor structure found in the Stellenbosch sample exhibited cross-cultural and -regional stability. The Chicago sample was not sufficiently large to enable separate CFA by sex and ethnicity. Sex invariance testing was therefore performed by collapsing across ethnicity and invariance testing for ethnicity was compared across the sexes. The CFA was first performed in the largest subsample of Chicago sample, Caucasian and non-Caucasian females (*n* = 582). The two-factor model provided a reasonable fit to the data (χ^2^(151) = 895.566, *p* < .001; RMSEA = .092 [90%*CI* = .086–.098]; CFI = .935; WRMR = 1.636) without freely estimating any error covariances. However, the factor intercorrelation was close to unity (ϕ = .979, [90%*CI* = .962–.995], *p* < .001). A one-factor model with seven freely estimated error covariances provide a more parsimonious representation of the pattern of covariances in the data (χ^2^(163) = 609.380, *p* < .001; RMSEA = .069 [90%*CI* = .063–.074]; CFI = .962; WRMR = 1.263). This one-factor model also provided a good fit to male participants, and in Caucasian and non-Caucasian participants when comparisons were collapsed across the sexes (Additional file 1: Table S5). The one-factor model (Additional file 1: Figure S2*)* was estimated in the combined Chicago sample (*N* = 827) and provided a reasonable fit (χ^2^(163) = 806.958, *p* < .001; RMSEA = .069 [90%*CI* = .064–.074]; CFI = .961; WRMR = 1.426).

### Unidimensional analyses

The discriminant validity of the two IAT factors in the Stellenbosch sample was evaluated by calculating the squared multiple correlation (SMC) and average variance extracted (AVE) [37]. The SMC was .687, whereas the AVE was .547 for factor one and .488 for factor two (.518 for both factors). These results indicate that these factors shared almost 69% of their variance but explained less than 52% of the variance in their respective items. Therefore, they shared more variance in common than they uniquely explained in IAT items indicating that combining these two factors into a unidimensional construct was warranted [29].

A bifactor model was fitted to the IAT data obtained from all Stellenbosch participants (*N* = 1661). Item 5 was introduced back into the analysis as it loaded on the general factor (λ = .285, *p* < .001). Items 8, 10, 13, and 18 did not exhibit statistically significant secondary loadings on the second group factor and were therefore constrained to zero. Model fit statistics were acceptable (χ^2^(155) = 1960.963, *p* < .001; RMSEA = .084 [90%*CI* = .080–.087]; CFI = .921; WRMR = 2.189), without including any error covariances. The first group factor did not have significant variance (σ^2^ = 23.446, *SE* = 16.735, *p* = .161). The ECV was calculated as .693 and the PUC .684, suggesting that collapsing the IAT into a unidimensional construct would introduce minimal parameter bias [59]. In combination with poor discriminant validity of the two-factor model as calculated by SMC and AVE, as well as non-significant residual variance in the first group factor, these findings indicated that a unidimensional model of the IAT in the Stellenbosch sample was appropriate.

### Latent class analysis of the IAT in the Stellenbosch sample

**Fig. 1:**
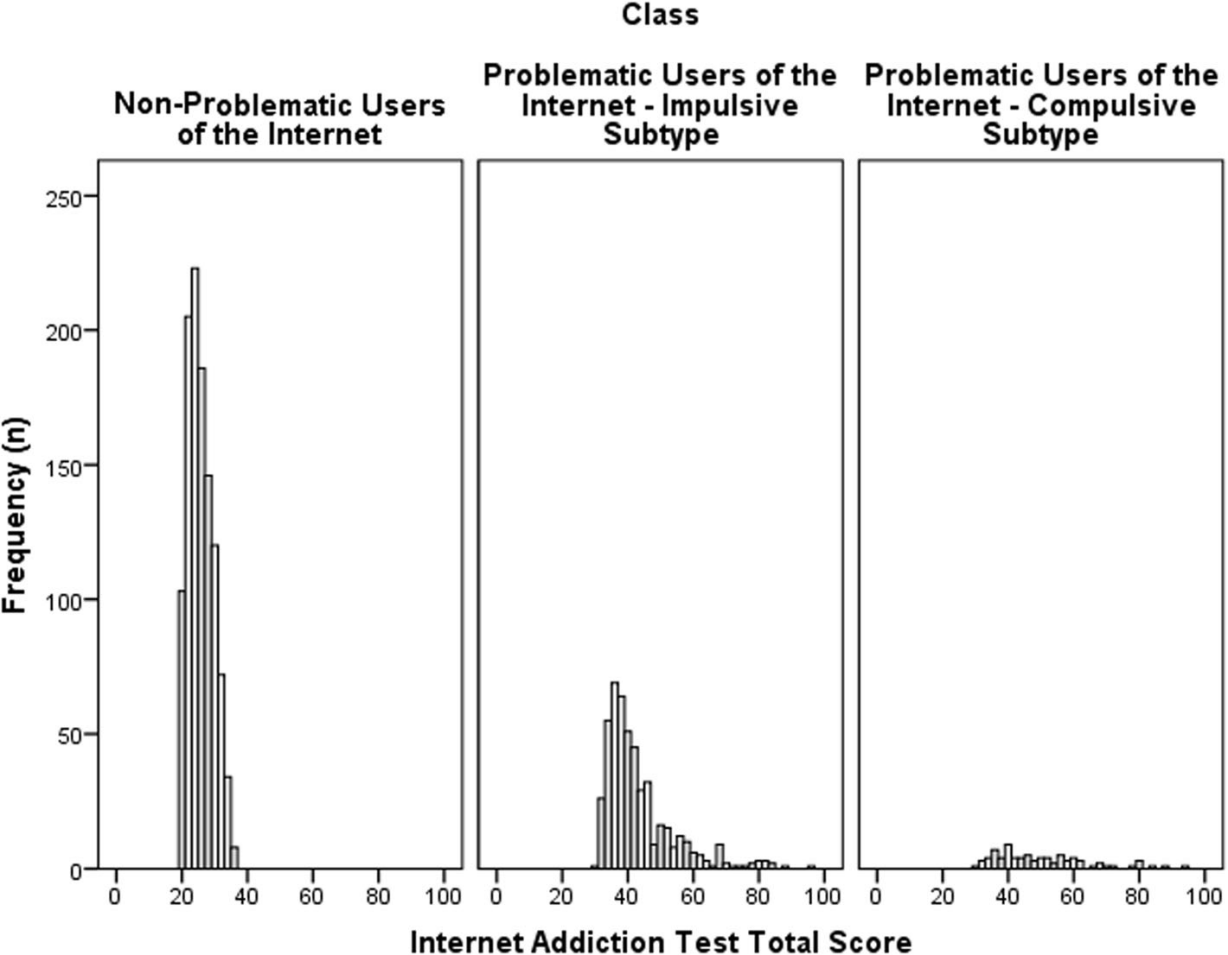
Distributions of total scores on the Internet Addiction Test for the three classes in the Stellenbosch dataset: 1) Non-Problematic Users of the Internet (*n* = 1097); 2) Problematic Users of the Internet Impulsive subtype (*n* = 483); and 3) Problematic Users of the Internet Compulsive subtype (*n* = 81)

The results of LCA conducted on the IAT in the Stellenbosch sample are summarized in Table 1. LCA indicated that a 2-class model provided the most parsimonious representation of the latent organization of the IAT data into groups. Detailed demographic and clinical characteristics of the two classes are summarized in Additional file 1: Tables S1 & S2. Class 1 (*n* = 564) was characterized by participants with an even distribution of probabilities for endorsing IAT items across the five response categories (hereafter termed ‘PUI’). It should be noted here that the definition PUI does not consider clinical thresholds, but rather LCA classes, which may or may not reflect clinical caseness. Class 2 (*n* = 1097) was characterized by participants with a high probability of endorsing “*Rarely*” for most IAT items and a low probability of endorsing any other response categories, reflecting an almost complete absence of Internet use problems (hereafter termed ‘Non-Problematic Users of the Internet’). The IAT total score distributions for these classes are shown in Fig*.* 1. The histogram in Fig. 1 reveals a log normal distribution with Non-Problematic Users of the Internet (NUI) characterised by IAT total scores falling below 30 and PUI participants exhibiting scores above 30 and located in the right tail of the distribution. Receiver Operating Characteristics were calculated for the IAT total scores and revealed excellent discriminating power (Area Under the Curve [AUC] = .994, *SE* = .001, [95%*CI* = .992–.996], *p* < .001), with .996 sensitivity and .896 specificity at an IAT total score of 30.5 for differentiating between NUI and PUI participants.

**Table 1:** Results of Latent Class Analysis of Internet Addiction Test and Problematic Use of the Internet Subtypes in the Stellenbosch Sample

Classes	Log Likelihood	BIC	Entropy	LMR	*p*
IAT Total					
1	−32,299.882^a^	65,192.978			
2	−29,177.004^a^	59,547.851	.906	6235.245	<.001
3	−28,257.598^a^	58,309.669	.901	1835.959	.746
PUI Subtypes					
1	− 7750.398^b^	15,887.233			
2	− 7376.133^b^	15,531.478	.830	746.628	<.001
3	− 7236.328^c^	15,644.641	.761	278.900	.759

*Note. LMR* Lo-Mendell-Rubin adjusted Likelihood Ratio Test when comparing the *k* to *k* – 1 class model; *p* = probability value for the Lo-Mendell-Rubin (LMR) adjusted Likelihood Ratio Test (LRT). IAT Total *N* = 1661. PUI Subtypes *N* = 564. ^a^Best loglikelihood values initially obtained using 160, 32 and then replicated using 320, 64 random starting value perturbations.^b^Best loglikelihood values initially obtained using 80, 16 and then replicated using 160, 32 random starting value perturbations.^c^Best loglikelihood value initially required 1280, 256 random starting value perturbations and then replication using 2560, 512 random starts

Between-group statistical comparisons based on χ^2^ test statistics and odds ratios (*OR*s), revealed statistically significant differences in the likelihood of PUI participants compared to NUI participants reporting symptoms consistent with all diagnostic categories covered by self-report, including GAD (χ^2^ (1) = 18.890, *p* < .001; *OR* = 2.003, *SE* = .305, [95%*CI* = 1.486–2.699]), SAD (χ^2^ (1) = 40.712, *p* < .001; *OR* = 3.346, *SE* = .589, [95%*CI* = 2.370–4.725]), ADHD (χ^2^ (1) = 29.136, *p* < .001, *OR* = 2.313, *SE* = .369, [95%*CI* = 1.692–3.161]), OCD (χ^2^ (1) = 20.182, *p* < .001, *OR* = 2.314, *SE* = .404, [95%*CI* = 1.643–3.260]), OCPD (χ^2^ (1) = 21.560, *p* < .001, *OR* = 1.901, *SE* = .283, [95%*CI* = 1.420–2.545]), and APD (χ^2^ (1) = 19.426, *p* < .001, *OR* = 1.804, *SE* = .244, [95%*CI* = 1.384–2.351]). PUI participants were also significantly more likely to report greater durations of use across all 13 online activities. There were also significant differences in group composition by sex (χ^2^ (1) = 10.448, *p* < .001), ethnicity (χ^2^ (1) = 28.414, *p* < .001), relationship status (χ^2^ (4) = 31.976, *p* < .001), and education χ^2^ (1) = 19.426, *p* < .001), with PUI participants more likely to be male (*OR* = 1.472, *SE* = .175, [95%*CI* = 1.165–1.859]), non-Caucasian (*OR* = 1.902, *SE* = .225, [95%*CI* = 1.508–2.397]), divorced / separated (*OR* = 3.431, *SE* = 1.183, [95%*CI* = 1.746–6.744]), and generally with lower levels of education. Comparison across classes revealed significant differences in age (χ^2^ (1) = 29.155, *p* < .001), and IAT total scores (χ^2^ (1) = 1369.228, *p* < .001), with PUI participants reporting higher mean age (*M* = 28.440, *SE* = .542) and IAT total scores (*M* = 44.525, *SE* = .510) compared to the age (*M* = 24.931, *SE* = .327) and IAT total (*M* = 24.748, *SE* = .119) scores of NUI. These results provide support for the separation of NUI (i.e. individuals reporting a relative absence of Internet use problems) and PUI (i.e. individuals with some degree of problematic Internet usage, along a continuum) participants.

Online activities and clinical measures were analyzed in the PUI class using follow-up LCA to determine if participants could be differentiated into PUI subtypes. Sex, ethnicity, education, and relationship status were entered as auxiliary variables. Data for use of the Internet for sport was removed from the analysis as low endorsement rates for 6–8 h (*n* = 4) and > 8 h (*n* = 1) were resulting in model identification problems. Data from online pornography use was missing for a higher proportion of PUI participants (*n* = 229, 40.6%). High levels of missingness can result in biased estimates using FIML and multiple imputation even under assumptions of MAR (Enders, 2010). Online pornography use was therefore excluded from the initial LCA to ensure the high percentage of missingness was not biasing model estimates and class enumeration. It was entered as an auxiliary variable to enable post hoc comparison across classes using the DCAT procedure. The results are summarized in Table 1. A two-class model provided significantly better fit to the data than one-class or three-class models. The majority of PUI participants (*n* = 483) were categorized into subclass 1, with the remainder in subclass 2 (*n* = 81). Subclass 1 PUI subjects were younger (*M* = 27.212, *SE* = .585) (χ^2^ (1) = 13.917, *p* < .001) and had significantly lower total scores on the IAT (*M* = 42.463, *SE* = .523; χ^2^ (1) = 16.963, *p* < .001) compared to the mean age (*M* = 33.517, *SE* = 1.505) and IAT score (*M* = 50.135, *SE* = 1.702) of subclass 2 participants. Subclass 2 PUI subjects reported higher of levels of Internet usage across all online activities compared to subclass 1 and were more likely to report symptoms consistent with diagnostic criteria for Social Anxiety Disorder (*OR* = 1.386 [*SE* = .508], *p* = .006), GAD (*OR* = 1.304 [*SE* = .469], *p* = .006); and OCPD (*OR* = 1.243 [*SE* = .431], *p* = .004). Subclass 1 PUI participants were significantly more likely to report ADHD symptoms (*OR* = 1.277 [*SE* = .431], *p* = .003).

There were no significant differences in composition between these subclasses in terms of sex (χ^2^ (1) = 1.4, *p* = .237) or relationship status (χ^2^ (4) = 7.419, *p* = .115). However, there was a higher proportion of non-Caucasian participants in subclass 2(52.5%) compared to subclass 1 (37%) (χ^2^ (1) = 4.652, *p* = .031). In addition, the participants in subclass 1 had significantly higher levels of education (χ^2^ (4) = 11.668, *p* = .020) compared to subclass 2 (41.7% versus 63.4% some college-level education). Thus, the first (subclass 1) was characterized by typically younger, more impulsive participants (PUI Impulsive) and the second (subclass 2) represented typically older, more compulsive participants (PUI Compulsive) with more severe PUI, potentially consistent with a theoretical shift towards compulsivity with chronicity. Comparisons across the two online activities included as auxiliary variables revealed significant differences in online sport (χ^2^ (5) = 17.468, *p* = .004, *n* = 479) and pornography use (χ^2^ (5) = 64.299, *p* < .001, *n* = 335) between the PUI classes. Only 8.1 and 17% of PUI subclass 1 participants reported at least 1–3 h of online sport and pornography viewing per day; whereas 34.6 and 47.3% of PUI subclass 2 participants reported this same level of use. The LCA was rerun including pornography and sport to ensure inclusion of these variables did not change class enumeration. A two-class model still provided the best fit as indicated by a significant likelihood ratio test for the one class model (LMR LRT = 844.295, *p* < .001) and a non-significant likelihood ratio test for the three-class model (LMR LRT = 306.100, *p* = 780). The IAT total score distributions for controls and each of these subclasses are shown in Fig. 1. ROC analysis indicated that total scores on the IAT could not be used to reliably differentiate these two PUI subtypes (AUC = .653, *SE* = .035, [95%*CI* = .585–.722], *p* < .01).

### Latent class analysis of the IAT in Chicago sample

**Fig. 2:**
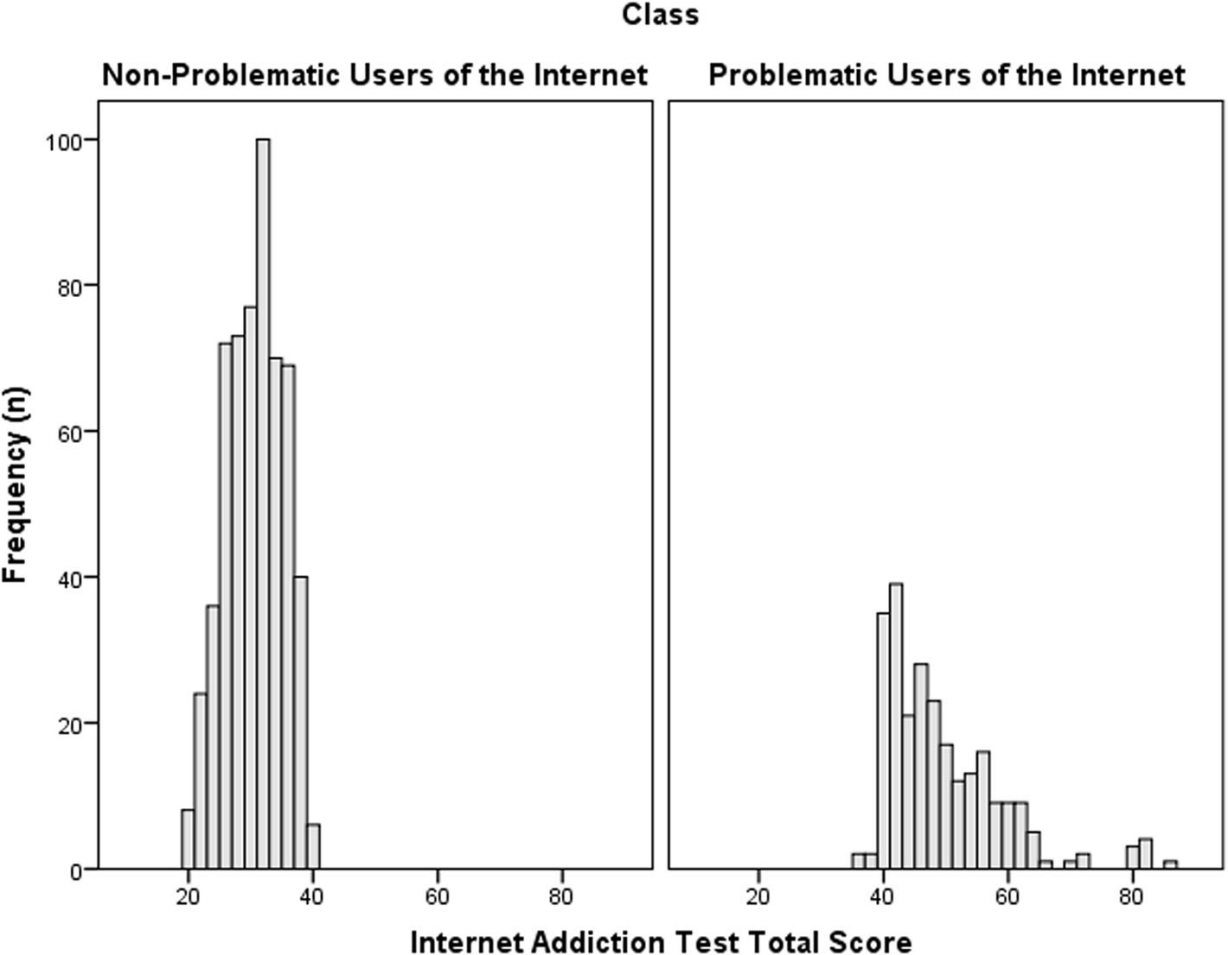
Distributions of total scores on the Internet Addiction Test for the two latent classes in the Chicago sample: 1) Non-Problematic Users of the Internet (*n* = 575) and 2) Problematic Users of the Internet (*n* = 252)

The results of LCA conducted on the IAT in the Chicago sample are summarized in Table 2. LCA indicated that a 2-class model differentiating between NUI (Class 1, *n* = 575) and PUI (Class 2, *n* = 252) participants provided the most parsimonious representation of the latent organization of the IAT data into groups (see Additional file 1: Results). The distributions of total IAT scores for each class are provided in Fig. 2. Demographic details of the groups are provided in Additional file 1: Table S3. As in the Stellenbosch sample, the NUI group were characterized by participants with a high probability of endorsing “*Rarely*” for most IAT items and a low probability of endorsing any other response categories. IAT total scores in this group ranged up to 40. The PUI group had a more even distribution of endorsement probabilities across item response categories.

**Table 2:** Results of Latent Class Analysis of the Internet Addiction Test and Problematic Use of the Internet Subtypes in the Chicago Sample

Classes	Log Likelihood	BIC	Entropy	LMR	*p*
IAT Total					
1	−17,677.464^a, b^	35,885.634			
2	−15,712.126^a, b^	32,492.383	.940	3923.374	<.001
3	−15,213.902^a, b^	32,033.359	.879	994.598	.760
PUI Subtypes					
1	− 4230.458^a^	8842.172			
2	− 4021.283^c^	8810.604	.815	417.271	.569
3	--3883.230^d,e^	8925.016	.832	282.587	.766

*Note.* LMR = Lo-Mendell-Rubin adjusted Likelihood Ratio Test when comparing the *k* to *k* – 1 class model; *p* = probability value for the Lo-Mendell-Rubin (LMR) adjusted Likelihood Ratio Test (LRT). IAT Total *N* = 1661. PUI Subtypes *N* = 564. ^a^Best loglikelihood values initially obtained using 80, 16 and then replicated using 160, 32 random starting value perturbations.^b^Problem of nonidentification for IAT item 4 threshold 4 in class 2 (PUI). ^c^Best loglikelihood values initially obtained using 160, 32 and then replicated using 320, 64 random starting value perturbations. ^d^Best loglikelihood values initially obtained using 1280, 256 and then replicated using 2560, 512 random starting value perturbations. ^e^Parameter estimation problems for Auction threshold 4 in Class 2, indicating possible model non-identification

Receiving Operating Characteristics were calculated for the IAT total scores and again revealed excellent discriminating power (AUC = .999, *SE* = .001, [95%*CI* = .997–1.000], *p* < .001), with .992 sensitivity and .963 specificity at an IAT total score of 37.5 for differentiating between the two latent classes. These results indicate that slightly higher IAT total scores were required to discriminate between classes in the Chicago, United States sample, in comparison to the Stellenbosch, South Africa sample. This was consistent with the observed higher mean scores in the Chicago (*M* = 35.83, *SD* = 10.77) compared to the Stellenbosch (*M* = 31.52, *SD* = 11.52) sample (*t* (2486) = 8.969, *p* = .001; *M* = 4.303, *SE* = .480, [95%*CI* = 3.36–5.24]). Online activities and clinical data were analyzed in the PUI group (*n* = 252) using a follow-up LCA to determine if participants could be differentiated into PUI subtypes. The results are summarized in Table 2. In contrast to the Stellenbosch sample, a one-class model provided a statistically significant better fit than a two-class model, suggesting that PUI subtypes could not be identified in this sample.

## Discussion

The field of PUI research has been hampered by conceptual ambiguity and methodological inconsistency [68]. Among the principal concerns has been the proliferation of measurement tools without clear consensus on the best approach to define and measure PUI [45]. The IAT emerged at the forefront of PUI research and remains a strong contender amongst existing instruments as a target of further development and refinement [42]. Here, we conducted a rigorous analysis of the IAT using a large dataset collected from two distinct geographical and cultural settings. The key finding was that PUI can be conceptualized as a unidimensional quasi-trait: that is to say, as a unipolar dimension in which most of the meaningful variance on the IAT is restricted to a subset of individuals experiencing difficulties regulating their Internet use. The majority of participants in the Stellenbosch (66%) and Chicago (69.5%) samples were characterized by a low probability of endorsing any IAT item response categories apart from ‘*Rarely*’, indicating an almost complete absence of Internet use problems. In contrast, a minority of participants from the Stellenbosch (34%) and Chicago (30.5%) samples exhibited a more even distribution of response category endorsement from ‘*Rarely*’ to ‘*Always*’ across the 20 IAT items and were classified as Problematic Users of the Internet.

This finding that PUI could be modeled as a unidimensional construct representing a single continuum of risk in this latent class is akin to prior findings in other areas of mental health, notably addictions [41]. However, in our analyses there appeared a clear demarcation point along a continuum of liability that could be used to differentiate problematic from non-problematic users of the Internet, albeit this demarcation is subject to future external validation. These findings may be consistent with a discontinuity hypothesis, in which there is posited a categorical and qualitative difference between individuals with and without PUI [56]. One possibility is that several, interrelated etiological processes with multiplicative effects increase over time and precipitate a transition from sub-clinical to clinically-significant levels of PUI, as has been proposed for other addictions [47]. This would be consistent with the log-normal distribution of IAT scores observed in the two samples examined in the current study [46]. Additionally, the advantage of modeling addictive behaviors as discrete latent classes for characterizing and predicting transitions in class membership over time have been previously demonstrated [43]. However, conclusions based on the application of a single instrument (i.e. the IAT) are necessarily limited and require validation with multiple, additional measures, collected over time (in order to model trajectories).

The IAT has received significant criticism due to the lack of empirically-defined cut-off scores [42]; ours is the first study to demonstrate that empirically-defined cut-offs can be determined, but that this is necessary for the precise population being examined. Total IAT scores performed exceptionally well in discriminating PUI from NUI participants identified via LCA, but the optimal threshold for defining these groups was different at each geographical location. The results indicate some degree of regional-specificity and cross-cultural instability of the IAT, a criticism that has been previously raised [42]. However, this issue is likely problematic for many psychiatric instruments including those used to assess PUI and related concepts, but is typically not considered in validation studies. Further research is required to directly compare the item-level psychometric properties between culturally-diverse groups to determine if the IAT is performing equivalently between heterogenous samples. Item Response Theory is a viable statistical approach to achieve this aim, because it places IAT scores on a common metric that enables direct group comparisons at the item and scale level [18, 60, 71]. Item Response Theory can also be used to refine and abbreviate the IAT and evaluate the properties and performance of the optimized scale in diverse samples (Edelden et al., 2007; Reise & Haviland, 2005). This issue is particularly important given previous criticisms of item redundancy and the pronounced psychometric instability of the IAT observed across studies. We suggest that the multiple factors found in previous psychometric studies of the IAT may be statistical artefacts associated with local item dependencies that are not related to the underlying unidimensional PUI continuum [13, 24, 38]. Factor instability across studies likely reflects changes in these statistical dependencies that are sample and study specific [42]. Our results also indicated that the IAT may be better suited to examining individuals with some degree of PUI, and that psychometric inconsistencies in prior work may have stemmed from PUI being a quasi-trait.

We did not find evidence for PUI subtypes based on profiling the online activities that they engaged in [55, 82]. The results showed rather that problematic engagement in various kinds of online activity increased uniformly with each other, suggesting an underlying commonality [4], rather than excessive Internet use being particularly related to only one specific candidate disorder such as Gaming Disorder, Gambling Disorder, or Compulsive Sexual Behaviour Disorder. The results are also consistent with theoretical assumptions that consider common aspects of the development and maintenance of specific types of problematic Internet use [8]. In the Stellenbosch sample, the first identified subtype represented participants that were younger, more likely to report impulsive symptoms (i.e. ADHD), and to report overall less time engaged in the 13 online activities. The second subtype reflected people who were older, more likely to report compulsive symptoms (i.e. OCPD), and to report spending generally more time engaged in all 13 online activities. These latent classes could also be viewed as defined by total time engaged in the Internet, which may reflect severity; but could also reflect a theoretical shift over time from a more ‘impulsive’ to a more ‘compulsive’ presentation [22]. This pattern might be consistent with the theoretically argued shift from experiences of gratification (more impulsively driven) to experiences of compensation (more compulsively driven) over time of problematic or even addictive Internet use [8]. These results suggest that compulsive rather than impulsive comorbid symptoms may in fact be more strongly linked to the more severe end of such Internet use. Interestingly, the impulsive and compulsive PUI subtypes could not be differentiated by summed scores on the IAT. Thus, collection of additional information regarding psychiatric comorbidity and duration of online activity appears necessary to discriminate between putative subtypes of PUI; hence, use of the IAT alone is inadequate for detection of such subtypes. If replicated, delineation of impulsive and compulsive subtypes of PUI may be clinically useful, in that impulsive and compulsive problems require very different treatment approaches, irrespective of PUI.

The present findings should be interpreted in the context of the methodological limitations of the study. These impulsive and compulsive PUI subtypes were not identified in the Chicago sample. The null finding may be due to the much smaller number of participants in this sample, which can make low-prevalence, yet substantively meaningful, classes difficult to identify [52]. Recruitment of large samples online has become a popular, useful, and widely accepted method for conducting large-scale studies in psychiatry [26]. Such an approach is particularly useful in dimensional psychiatry, where it is assumed that mental health symptoms are not merely present or absent but extend across the full spectrum of severity from non-clinical, sub-clinical, and clinical levels [23, 39]. Dimensional approaches also afford increased statistical power for studying the relationships of interest [40]. Nevertheless, the use of a self-selected sample in the current study, rather than a selected, representative or stratified sample introduces limitations to the robustness and generalizability of the findings. Self-report data may be subject to systematic biases and common method variance, and self-report measures are not as widely validated as in-person clinical assessments using the same or comparable instruments. We used modules obtained from the MINI that were modified for self-report to measure symptoms consistent with OCD, SAD, GAD, and APD, an approach that has not yet been externally validated. This method may not have yielded results with the same clinical accuracy as a diagnostic interview. Future work would benefit from obtaining information from multiple informants, as well as from objective sources including clinical interview, as a way of surmounting common method variance [57, 58]. Information regarding a broader range of psychiatric symptoms and other addictive disorders would be particularly useful for expanding our understanding of PUI in the context of existing nosologies and may assist in more accurately identifying subtypes using LCA [73, 79]. Follow-up studies are needed that use rigorous in-person clinical assessment, rather than relying on online data, which is likely to be clinically less precise. It cannot be guaranteed that the findings will be representative of PUI more broadly. Nevertheless, we were able to replicate the unidimensional quasi-trait structure of PUI across independent, geographically-diverse samples, suggesting this finding was relatively robust. Lastly, the IAT of course represents just one scale used to explore PUI, and it would be valuable to apply approaches herein to the evaluation of other scales in future work. This seems important since the items of the IAT, and many other instruments for PUI and related constructs, were not designed for differentiating specific subtypes based on impulsive and compulsive features.

## Conclusions

The current study contributes several important insights into the nature, classification, and measurement of PUI. First, we showed PUI to be a unidimensional quasi-trait, with clinically meaningful variance in the severity of symptoms restricted to a subset of people. Thus, the IAT measures a continuum of Internet use problems, but is psychometrically useful only in a minority (albeit a sizeable proportion) of a given community-based sample such as this. This issue applies to many scales in psychiatry (e.g. depression scales) but is seldom considered in terms of the effects of this property of psychometric stability. There was no evidence for subtypes of PUI as a function of another primary addictions based on online activities endorsed (e.g. gambling, streaming, gaming …). Rather, analyses provided initial support for severity subtypes, with a compulsive subtype being associated with more frequent Internet use. These data indicate that the measurement properties of IAT items cannot be directly compared between geographically and culturally diverse samples, but rather require study-specific (or perhaps culture-specific) calibration. The findings of the current study can be used to concentrate further research on individuals at the risk-end of the PUI continuum, and we hope may lead to refinements to scales that overcome psychometric limitations, in order to measure Internet use problems with greater levels of confidence and precision.

## Supplementary information

**Additional file 1.** Supplementary Material.

## Data Availability

The datasets generated and analysed during the current study are not publicly available since participants did not consent for external data sharing.

## Abbreviations

ADHD: Attention-deficit hyperactivity disorder
APD: Avoidant personality disorder
AUC: Area under the curve
CFA: Confirmatory factor analysis
DSM: Diagnostic and statistical manual
EFA: Exploratory factor analysis
FDR: False discovery rate
GAD: Generalized anxiety disorder
IAT: Internet addiction test
ICD: International classification of diseases
LCA: Latent class analysis
LMR: Lo-Mendell-Rubin
LRT: Likelihood ratio test
MIDI: Minnesota impulse disorders inventory
MINI: Mini international neuropsychiatric inventory
OCD: Obsessive-compulsive disorder
PUI: Problematic usage of the internet
RMSEA: Root mean squared error of approximation
SAD: Social anxiety disorder
WLSMV: Weighted least squares means and variance
WRMR: Weighted root mean residual
