# Supplementary material for: Problematic use of the Internet is a unidimensional quasi-trait with impulsive and compulsive subtypes
Source: BMC Psychiatry. 2019 Nov 8;19:348. doi: 10.1186/s12888-019-2352-8 (PMC6839143; doi:10.1186/s12888-019-2352-8)
Supplement: Supplementary file 1 — Additional file 1. Supplementary Material. [file 12888_2019_2352_MOESM1_ESM.docx]

**Supplementary Material**

**Methods**

**Missing Data**

There were no missing data or out-of-range values for the IAT or demographic variables in the Stellenbosch sample. Data was missing for 277 (16.7% ) of the Stellenbosch sample for 8/12 online activities; *n* = 278 for Streaming, Messaging, and Shopping; and *n* = 536 (32.3%) for online pornography. In the PUI class, 85 (15.1%) participants were missing data for 11 of the online activities; 86 (15.2%) missing for online shopping, and 229 (40.6%) for online pornography. Data was also missing for self-reported symptoms consistent with Social Anxiety (*n* = 1, 0.2%), Obsessive-Compulsive Personality Disorder (*n* = 81, 14.4%), Avoidant Personality Disorder (*n* = 82, 14.5%), and Attention Deficit Hyperactivity Disorder (*n* = 99, 17.6%). Item response data were missing on the IAT for *n* = 63 (7.1%) participants from the Chicago dataset, such that a subset (*n* = 827) were analysed using LCA to delineate Non-Problematic from Problematic Users of the Internet. There were no missing data or out-of-range values for the demographic variables in the Chicago dataset other than 8 participants missing data for relationship status; 127 (15.4%) of participants were missing data for online activities, Generalized Anxiety Disorder, and Attention Deficit Hyperactivity Disorder symptoms. In the Chicago PUI subsample, 42 (16.7%) participants were missing data for online activities; self-reported symptoms of Generalized Anxiety Disorder and Attention Deficit Hyperactivity Disorder. A smaller proportion were missing data for self-reported symptoms consistent with Social Anxiety (*n* = 35, 13.9%) and Obsessive-Compulsive Personality Disorder (*n* = 40, 15.9%). No information was obtained for Avoidant Personality Disorder (APD) symptoms from participants in the Chicago sample. Bivariate contingency tables revealed statistically significant correlations between variables with missing data and covariates included in the LCA models, indicating that the assumptions of Missing At Random (MAR) were satisfied (Enders, 2010). Full Information Maximum Likelihood (FIML) with expectation maximization (EM) was used for model estimates in LCA where variables with missing data were included as covariates in the model (Muthén & Muthén, 1998 - 2012). Model estimates using FIML are unbiased when the assumptions of MAR are met (Enders, 2010). However, high levels of missingness can result in biased estimates using FIML and multiple imputation even under assumptions of MAR (Enders, 2010).Thus, the online pornography variable, which exceeded 40% missingness, was excluded from the LCA model to ensure inclusion did not bias model estimation and class enumeration. Default listwise deletion was used for comparison between latent classes across auxiliary variables using the DCAT and BCH procedures (Asparouhov and Muthén, 2014, Lanza et al., 2013).

**Supplementary Methods Detail A**

The -2*LL and BIC values were initially generated for 1 – 10 class models and plotted to identify a smaller range of plausible models. From this smaller range of models, the best log likelihood values were obtained for each number of classes tested using an initial number of random starting value perturbations (Nylund et al., 2007). The model was then rerun with double the number of random starting value perturbations to ensure that the analyses did not converge on local maxima in estimating the best log likelihood value. Once the best log likelihood was replicated, each model was rerun to obtain the LMR adjusted LRT by using the seed that resulted in the best log likelihood value specified as the starting value instead of random starts (Asparouhov, & Muthén, May 22, 2012).

**Results**

**Supplementary Table S1. Descriptive Statistics of Normal and Problematic Users of the Internet in the Stellenbosch Sample**

| **Class** | ***N*** | **Age** | | **Sex**^1^ | | **Ethnicity**^2^ | | **Education** | | | | | **IAT** | |
| --- | --- | --- | --- | --- | --- | --- | --- | --- | --- | --- | --- | --- | --- | --- |
|  |  | *M*  *(SD)* | Range | Female | Male | Cauc | Non-Cauc | < HS | HS Grad | < Coll | Coll Grad | Coll + | *M*  *(SD)* | Range |
| NUI | 1097 (66%) | 25.01 (10.56) | 17 - 76 | 704 (64.2%) | 388 (35.4%) | 804 (73.3%) | 293 (26.7%) | 0 | 162 | 182 | 490 | 263 | 25.21  (3.73) | 20 - 36 |
| PUI | 564 (34%) | 28.31  (12.39) | 17 - 88 | 316 (56.0%) | 244 (43.3%) | 340 (60.3%) | 224 (39.7%) | 1 | 68 | 160 | 226 | 109 | 43.80 (11.63) | 29 - 96 |
| Total | 1661 | 26.13  (11.32) | 17 - 88 | 1020  (61.4%) | 632  (38.0%) | 1144  (68.9%) | 517  (31.1%) | 1 | 230 | 342 | 716 | 372 | 31.52  (11.52) | 20 - 96 |

*Note. N* = Number of participants. ^1^Counts and percentages do not represent the total as a small portion of the sample identified as transgender. ^2^For the purposes of classification, ethnicity was recorded dichotomously as Caucasian and Non-Caucasian. *M* = Mean. *SD* = Standard Deviation. Cauc = Caucasian. Non-Cauc = Non-Caucasian. < HS = High school not completed. HS Grad = Graduated High school. < Coll = College not completed. Coll Grad = Graduated College. + Coll = College post-Graduate studies. IAT = Internet Addiction Test. NUI = Non-Problematic Users of the Internet. PUI = Problematic Users of the Internet.

**Supplementary Table S2. Descriptive Statistics of for Two Groups of Problematic Users of the Internet in the Stellenbosch Sample**

| **Class** | ***N*** | **Age** | | **Sex**^1^ | | **Ethnicity**^2^ | | **Education** | | | | | **IAT** | |
| --- | --- | --- | --- | --- | --- | --- | --- | --- | --- | --- | --- | --- | --- | --- |
|  |  | *M*  *(SD)* | Range | Female | Male | Cauc | Non-Cauc | < HS | HS Grad | < Coll | Coll Grad | Coll + | *M*  *(SD)* | Range |
| Imp | 483 (85.6%) | 27.50 (12.14) | 17 - 88 | 276 (57.1%) | 203 (42.0%) | 299 (61.9%) | 184 (38.1%) | 0 | 57 | 127 | 200 | 99 | 42.81 (10.78) | 30 - 96 |
| Comp | 81 (14.4%) | 33.17 (12.84) | 18 - 62 | 40 (49.4%) | 41 (50.6%) | 41 (50.6%) | 40 (49.4%) | 1 | 11 | 33 | 26 | 10 | 49.72 (14.52) | 29 - 94 |

*Note. N* = Number of participants. ^1^Counts and percentages do not represent the total as a small portion of the sample identified as transgender. ^2^For the purposes of classification, ethnicity was recorded dichotomously as Caucasian and Non-Caucasian. *M* = Mean. *SD* = Standard Deviation. Cauc = Caucasian. Non-Cauc = Non-Caucasian. < HS = High school not completed. HS Grad = Graduated High school. < Coll = College not completed. Coll Grad = Graduated College. + Coll = College post-Graduate studies. IAT = Internet Addiction Test. IMP = Impulsive Problematic Users of the Internet. Comp = Compulsive Problematic Users of the Internet.

**Supplementary Table S3. Descriptive Statistics of Normal and Problematic Users of the Internet in the Chicago Sample**

| **Class** | ***N*** | **Age** | | **Sex**^1^ | | **Ethnicity**^2^ | | **Education** | | | | | **IAT** | |
| --- | --- | --- | --- | --- | --- | --- | --- | --- | --- | --- | --- | --- | --- | --- |
|  |  | *M*  *(SD)* | Range | Female | Male | Cauc | Non-Cauc | < HS | HS Grad | < Coll | Coll Grad | Coll + | *M*  *(SD)* | Range |
| NUI | 575 (69.5%) | 36.75 (14.59) | 18 - 76 | 411 (71.5%) | 157 (27.3%) | 446 (77.6%) | 129 (22.4%) | 7 | 47 | 188 | 216 | 117 | 30.10 (4.52) | 20 - 40 |
| PUI | 252 (30.5%) | 33.74 (13.81) | 16 - 77 | 171 (67.9%) | 77 (30.6%) | 170 (67.5%) | 82 (32.5%) | 7 | 29 | 96 | 82 | 38 | 48.89 (9.42) | 36 - 85 |
| Total | 827 | 35.83 (14.42) | 16 - 77 | 582 (70.4%) | 234 (28.3%) | 616 (74.5%) | 211 (25.5%) | 14 | 76 | 284 | 298 | 155 | 35.83 (10.77) | 20 - 85 |

*Note. N* = Number of participants. ^1^Counts and percentages do not represent the total as a small portion of the sample identified as transgender. ^2^For the purposes of classification, ethnicity was recorded dichotomously as Caucasian and Non-Caucasian. *M* = Mean. *SD* = Standard Deviation. Cauc = Caucasian. Non-Cauc = Non-Caucasian. < HS = High school not completed. HS Grad = Graduated High school. < Coll = College not completed. Coll Grad = Graduated College. + Coll = College post-Graduate studies. IAT = Internet Addiction Test. NUI = Non-Problematic Users of the Internet. PUI = Problematic Users of the Internet.

**Supplementary Results - Confirmatory Factor Analysis with Invariance Testing of the Internet Addiction Test**

The IAT 2-factor model estimated in Caucasian female participants from the Stellenbosch sample based on the results of Exploratory Factor Analysis is displayed in *Figure S1* and provided a reasonable overall fit (χ^2^(139) = 631.052, *p* <.001; RMSEA = .072 [90%*CI* =.066 - .077]; CFI = .951; WRMR = 1.417). Item loadings were generally high with no cross-loadings. The factor intercorrelation was also strong and statistically significant (ϕ = .803, *p* < .001). This two-factor model was cross-validated in non-Caucasian females, as well as Caucasian and non-Caucasian males. Model fit statistics and the results of invariance testing as provided in Table S4. Latent means for Factor 1 and Factor 2 were significantly in higher in non-Caucasian females (*t*(687) = 4.073, *p* < .001; *t*(687) = 3.230, *p* = .001) and males (*t* (687) = 4.271, *p* < .001; *t* (687) = 2.583, *p* = .010) compared to their Caucasian counterparts. Latent means for the two IAT factors were also significantly higher in males compared to females ((*t* (1649) = 3.629, *p* < .001; *t* (1649) = 2.909, *p* = .004). However, there were no significant sex-related differences in factor variances (Δχ^2^ (2) = 4.767, *p* = .092; ΔCFI = .011; ΔRMSEA = .008) or the factor covariance (Δχ^2^ (1) = .755, *p* = .385; ΔCFI = .007; ΔRMSEA = .002). These results suggest that the latent structure of PUI and the measurement properties of the IAT were largely similar between the sexes and Caucasian and non-Caucasian participants in the Stellenbosch sample. Thus, differential item functioning was largely absent when comparing across naturally occurring groups based on sex and ethnicity. The only observed differences were in latent means, factor variances, and covariances.

**Supplementary Results - The results of LCA conducted on the IAT in the Chicago sample**

Results indicated estimation problems with threshold four of item 19 and then item 20, suggesting these parameters were not properly identified. These two IAT items were removed and the LCA models re-estimated to ensure the results were reliable. A 2-class model (2*-LL = -14469.999; BIC = 29900.644; *E* = .925; LMR LRT = 3311.813, p < .001) still provided a better fit to the data compared to one-class (2*-LL = --16129.329; BIC = 32735.622) and three-class (2*-LL = -14040.645; BIC = 29525.618; E = .865; LMR LRT = 856.936, p = .309) models. Changes in class separation and class assignment to the PUI (n = 266) and NUI (n = 561) groups after removal of IAT items 19 and 20 were small. These reanalyses suggested that the parameter estimation problems for these item thresholds were not significantly biasing the results.

**Supplementary Table S4. Results of Confirmatory Factor Analysis and Invariance Testing for the Internet Addiction Test for Caucasian and Non-Caucasian Males and Females in the Stellenbosch Sample**

|  | Model | *df* | *χ*^2^ | *p* | RMSEA (90%*CI*) | CFI | WRMR | Δ*df* | Δ*χ*^2^ | *p* | ΔCFI | ΔRMSEA |
| --- | --- | --- | --- | --- | --- | --- | --- | --- | --- | --- | --- | --- |
| Caucasian  Females | Two Factor  Model | 141 | 516.520 | <.001 | .077 (.070 - .084) | .926 | 1.310 |  |  |  |  |  |
| Non-Caucasian Females | Configural Invariance | 289 | 1144.179 | <.001 | .076 (.072 - .081) | .944 | 1.944 |  |  |  |  |  |
|  | Partial Measurement Invariance^1^ | 359 | 1098.010 | <.001 | .064 (.059 - .068) | .952 | 2.220 | 70 | 170.401 | <.001 | .008 | .012 |
| All Females | Two Factor  Model | 133 | 832.024 | <.001 | .072 (.067 - .077) | .953 | 1.531 |  |  |  |  |  |
| Caucasian Males | Two Factor  Model | 141 | 516.520 | <.001 | .077 (.070 - .084) | .926 | 1.310 |  |  |  |  |  |
| Non-Caucasian Males | Configural Invariance | 298 | 899.987 | <.001 | .080 (.074 - .086) | .928 | 1.760 |  |  |  |  |  |
|  | Measurement Invariance | 383 | 944.988 | <.001 | .068 (.063 - .074) | .933 | 2.143 | 85 | 204.352 | <.001 | .005 | .012 |
| All Males | Two Factor  Model | 141 | 652.991 | <.001 | .076 (.070 - .082) | .939 | 1.406 |  |  |  |  |  |
| Sex Invariance | Configural Invariance | 288 | 1492.063 | <.001 | .071 (.068 - .075) | .949 | 2.127 |  |  |  |  |  |
|  | Partial Measurement Invariance ^2^ | 371 | 1325.073 | <.001 | .056 (.052 - .059) | .959 | 2.451 | 83 | 209.627 | <.001 | .010 | .015 |

*Note.*^1^ The factor loadings and thresholds of items 2, 3, and 6 were freely estimated. ^2^ The factor loadings and thresholds of items 10 and 14 were freely estimated. Delta degrees of freedom (Δ*df*) and delta chi square (Δ*χ*^2^) obtained using the ‘DIFFTEST’ option of Mplus (Muthén and Muthén, 1998 - 2012). Total N **=** 1661; Females N = 1020 (Caucasian n = 689; Non-Caucasian n = 330); Males N = 632 (Caucasian n = 449; Non-Caucasian n = 183).


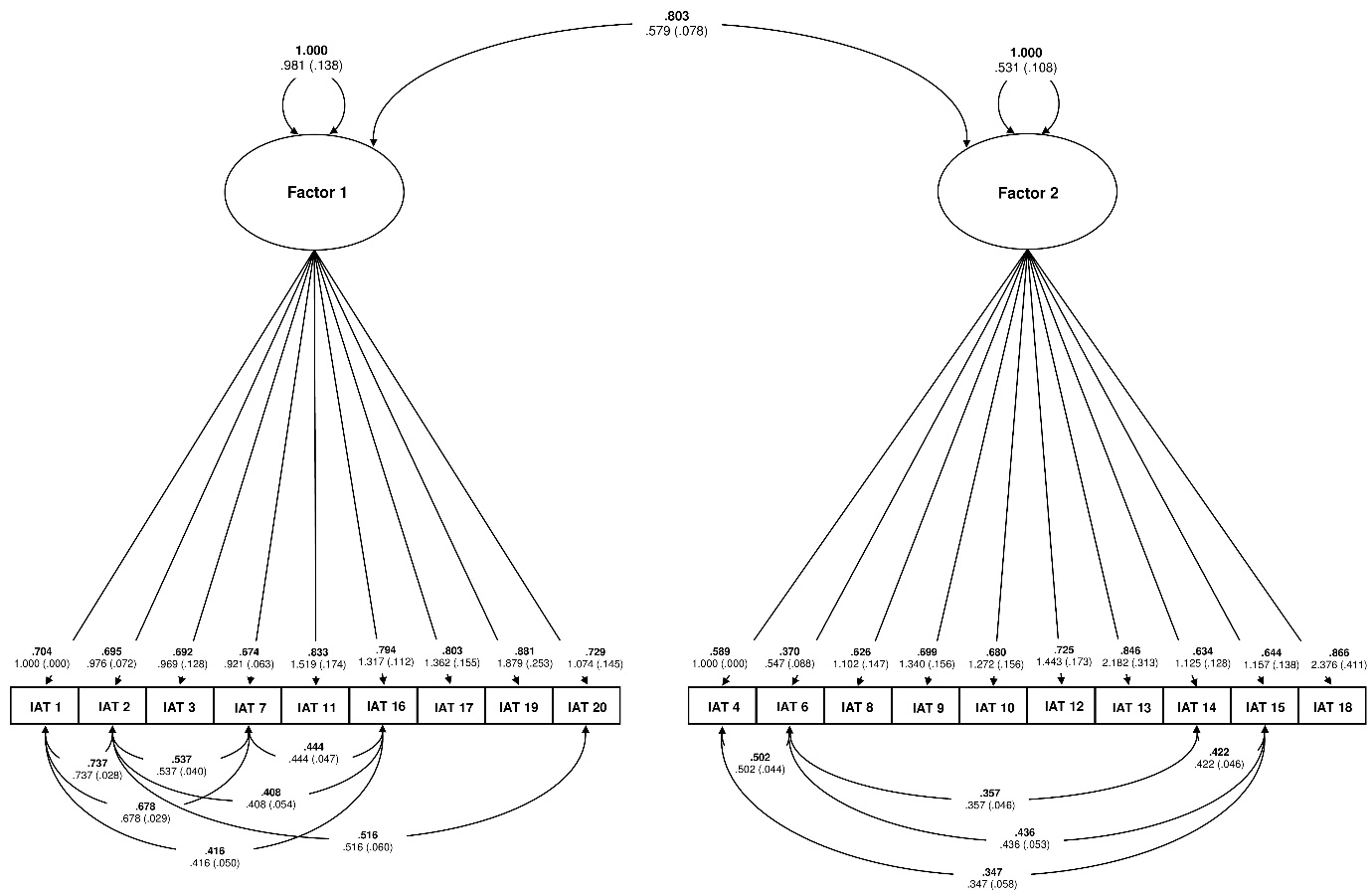


**Supplementary Figure S1. Two-Factor model of the Internet Addiction Test in female Caucasian participants from the South African sample.**

*Note.* Model fit was χ^2^(139) = 631.052, *p* <.001; RMSEA = .072 [90%*CI* =.066 - .077]; CFI = .951; WRMR = 1.417. Standardized parameter estimates are bold type face with unstandardized estimates below and standard errors in brackets. All parameters were significant at *p* < .001. Error covariances were all significant when corrected for multiple comparisons using the Benjamini-Hochberg False Discovery Rate (*q* = .05).

**Supplementary Table S5.**

**Results of Confirmatory Factor Analysis for the Internet Addiction Test for Females, Males, Caucasian, and Non-Caucasian Participants in the Chicago Sample**

|  | Model | *df* | *χ*^2^ | *p* | RMSEA (90%*CI*) | CFI | WRMR | *n* |
| --- | --- | --- | --- | --- | --- | --- | --- | --- |
| Females | Two-Factor | 151 | 895.566 | <.001 | .092 (.086 - .098) | .935 | 1.636 | 582 |
|  | One-Factor^1^ | 163 | 609.380 | <.001 | .069 (.063 - .074) | .962 | 1.263 |  |
| Males | One-Factor^1^ | 163 | 301.803 | <.001 | .060 (.050 - .071) | .974 | .905 | 234 |
| Caucasian | One-Factor^1^ | 163 | 637.780 | <.001 | .069 (.063 - .074) | .959 | 1.295 | 616 |
| Non-Caucasian | One-Factor^1^ | 164 | 637.780 | <.001 | .066 (.055 - .077) | .973 | .931 | 211 |
| Whole Sample | One-Factor^1^ | 163 | 806.958 | <.001 | .069 (.064 - .074) | .961 | 1.426 | 827 |

*Note.*^1^ Includes freely estimated error covariances, all significant when adjusted for multiple *post hoc* comparisons (B-H FDR *q* = .05).


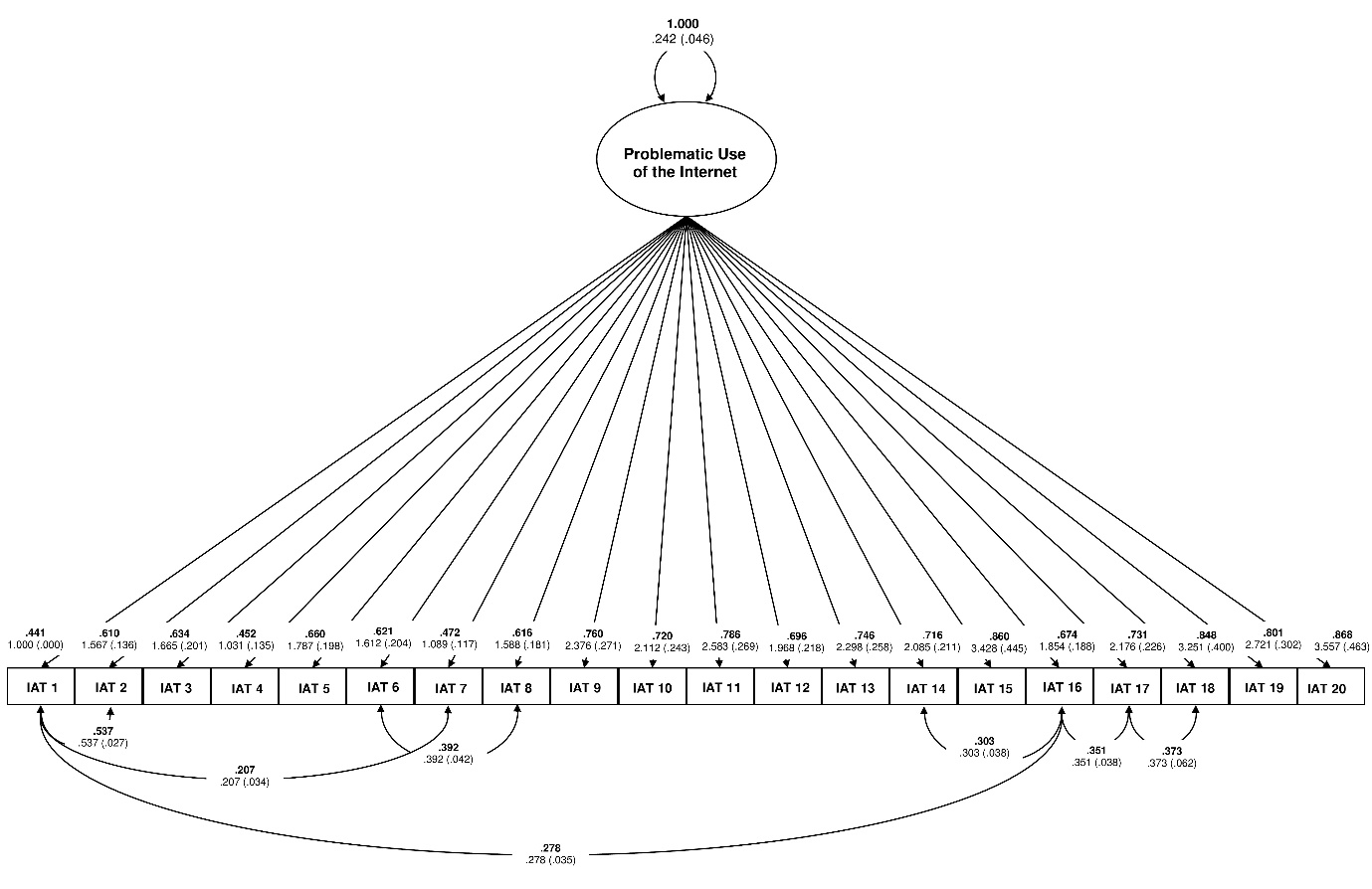


**Supplementary Figure S2. One-Factor model of the Internet Addiction Test in the United States sample.**

*Note.* *N* = 827. Model fit (χ^2^(163) = 806.958, *p* <.001; RMSEA = .069 [90%*CI* =.064 - .074]; CFI = .961; WRMR = 1.426). Standardized parameter estimates are bold type face with unstandardized estimates below and standard errors in brackets. All parameters were significant at *p* < .001. Error covariances were all significant when corrected for multiple comparisons using the Benjamini-Hochberg False Discovery Rate (*q* = .05).


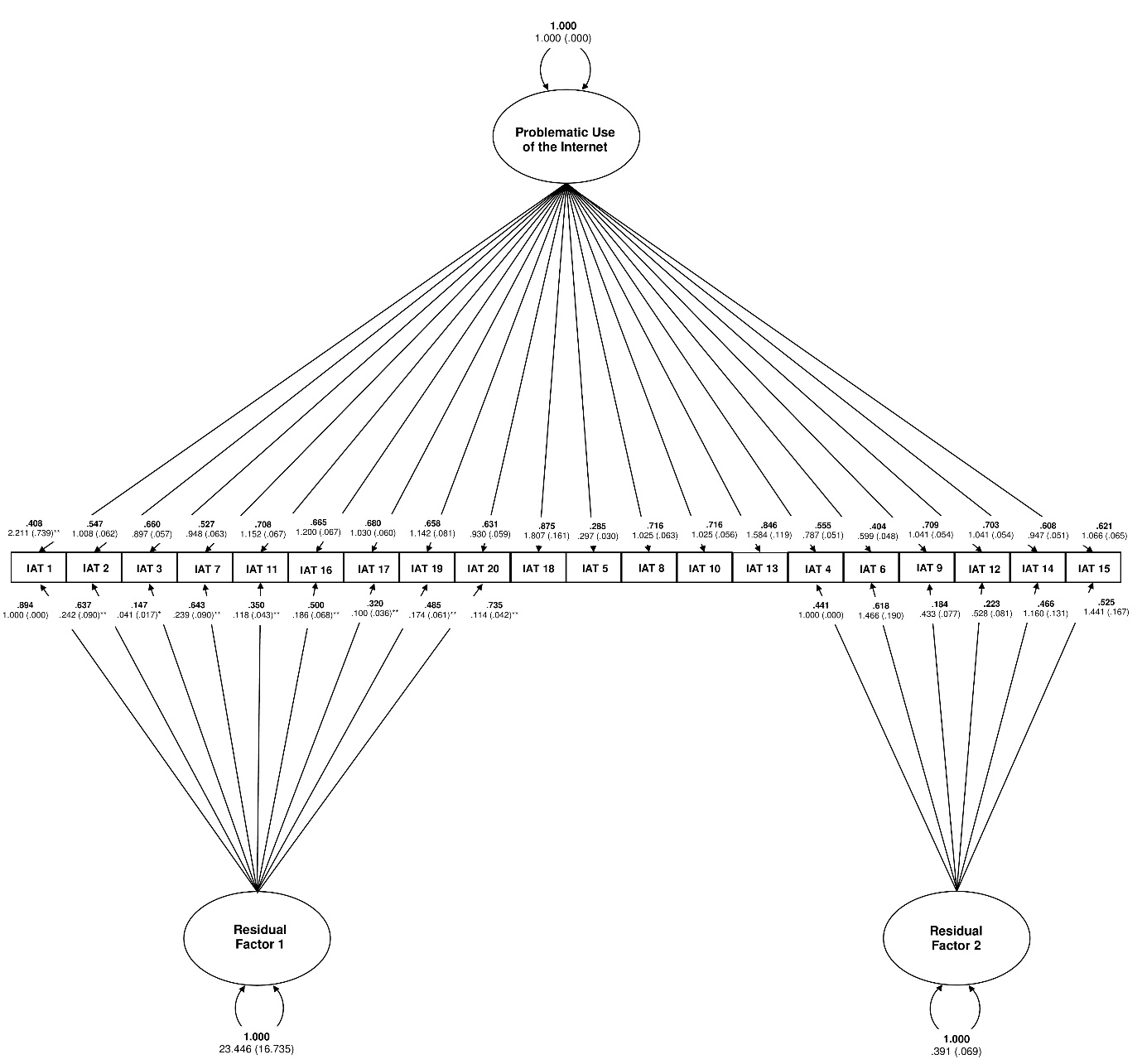


**Supplementary Figure S3. Bi-Factor model of the Internet Addiction Test in participants from the Stellenbosch sample.**

*Note.* Bifactor model of the Internet Addiction Test with a general factor (top) capturing common variance shared across all IAT items and representing a unidimensional liability to Problematic Use of the Internet, and the bottom two group factors capturing residual shared variance amongst a subset of items. *N* = 1661. Model fit was χ^2^(155) = 1960.963, *p* <.001; RMSEA = .084 [90%*CI* =.080 - .087]; CFI = .921; WRMR = 2.189. Standardized parameter estimates are bold type face with unstandardized estimates below and standard errors in brackets. All parameters were significant at *p* < .001, except the variance of Residual Factor 1, which was non-significant (*p* > .05), and where otherwise indicated. ***p* <.01. Error covariances were all significant when corrected for multiple comparisons using the Benjamini-Hochberg False Discovery Rate (*q* = .05).
